# The EFSA Health Claim on Olive Oil Polyphenols: Acid Hydrolysis Validation and Total Hydroxytyrosol and Tyrosol Determination in Italian Virgin Olive Oils

**DOI:** 10.3390/molecules24112179

**Published:** 2019-06-10

**Authors:** Maria Bellumori, Lorenzo Cecchi, Marzia Innocenti, Maria Lisa Clodoveo, Filomena Corbo, Nadia Mulinacci

**Affiliations:** 1 Department of NEUROFARBA, University of Firenze, Via Ugo Schiff 6, 50019 Sesto F.no (Firenze), Italy and Multidisciplinary Centre of Research on Food Sciences (M.C.R.F.S.-Ce.R.A.); maria.bellumori@unifi.it (M.B.); lo.cecchi@unifi.it (L.C.); marzia.innocenti@unifi.it (M.I.); 2 Interdisciplinary Department of Medicine, University of Bari, Piazza Giulio Cesare, 11-70124 Bari, Italy; marialisa.clodoveo@uniba.it; 3 Department of Pharmacy-Pharmaceutical Sciences, University Aldo Moro-Bari, Via Orabona, 4, 70124 Bari, Italy

**Keywords:** acidic hydrolysis, validation, oleuropein, ligstroside, phenolic compounds, secoiridoids, HPLC/DAD, European Commission Regulation 432/2012

## Abstract

The health claims of olive oil represent an important marketing lever in raising the willingness to pay for a product, but world producers of extra virgin olive oil (EVOO) do not take advantage of it because there are still obstacles to their use. Among these, one issue is the lack of an official method for determination of all free and linked forms derived from secoiridoidic structures of hydroxytyrosol and tyrosol. In this study, different acidic hydrolytic procedures for analyzing the linked forms were tested. The best method was validated and then applied to more than 100 EVOOs. The content of oleuropein and ligstroside derivatives in EVOOs was indirectly evaluated comparing the amount of phenols before and after hydrolysis. After acidic hydrolysis, a high content of total tyrosol was found in most of the EVOOs. The use of a suitable corrective factor for the evaluation of hydroxytyrosol allows an accurate determination only using pure tyrosol as a standard. Further knowledge on the concentration of total hydroxytyrosol will assist in forecasting the resistance of oils against aging, its antioxidant potential and to better control its quality over time.

## 1. Introduction

The new lifestyle habits and the relative spending patterns linked to a growing demand for well-being are shaping growing consumption trends for health foods. The tendency to exalt the health virtues of a product, and the awareness of the value attributed by the consumer to these characteristics, has enormously influenced advertising communications. There are cases of misleading advertising which are based on health-related information without scientific basis and capable of conditioning consumer choices and eating habits. In response, the European Parliament approved regulation 1924/2006 [1] which governs nutrition and health claims provided on food products. The claims must therefore be understandable for the consumer and must demonstrate, based on scientific evidence, that the nutrient has a nutritional or beneficial effect.

The Regulation (EU) No. 432/2012 [2] of the European Commission of May 16, 2012 has defined a list of permitted health claims and the European Register of Nutrition and Health Claims (EU Register of Nutrition and Health Claims) has provided food reports on all the authorized health indications, conditions and restrictions of use, as well as unauthorized health claims and the reasons for their inapplicability. The permitted health claims for olive oil are relative to olive oil polyphenols, oleic acid, vitamin E and monounsaturated and/or polyunsaturated fatty acids (see Supplementary Material).

The best extra virgin olive oils are obtained by early harvesting the drupes, working the product promptly, reducing the process water as much as possible, and limiting the heating of the olive pasta to be able to maintain a content of bioactive phenolic substances useful for the application of the health claim [3,4,5,6,7,8]. The interesting aspect that can arise from scientific evidence to date is that the application of all the aforementioned good practices also positively influence the other parameters required by the law for the classification of virgin olive oils (acidity, peroxide value, K_232_ and K_270_). The health claim regarding polyphenols, therefore, lends itself to being a useful legislative instrument for the segmentation of the category of extra virgin olive oil. It allows the consumer to recognize the health claim approved by EFSA (European Food Safety Authority) on the label of the bottle, and the highest quality segment within the product category of extra virgin olive oil [9,10]. The current product classification of olive oils, conceived in 1991, is obsolete and insufficient to adequately describe the qualitative differences of the (extra virgin olive oils) EVOOs on the market. As a result, the polyphenol claim becomes a useful differentiation tool for the consumer to attribute a premium price to the best products. It is interesting to note that no more than 10% of bottled oils available on the market have a suitable phenolic content for the application of the health claim [11]. This datum opens a reflection on the direction of which efforts should be directed to support the European olive oil supply chain, guaranteeing adequate profitability to producers and millers.

The problem of accurate determination of the secoiridoidic compounds, which are the main polyphenols in virgin olive oil, is to date unsolved, and several reasons are behind this issue. Firstly, the two main secoiridoidic glycosides present in the olive fruit, namely oleuropein and ligstroside, are rapidly transformed into several de-glycosilated derivatives during milling by the action of native β-glucosidases. The aglycones are then furtherly transformed by enzymatic and chemical reactions, leading to the opening of the secoiridoidic ring and the formation of several aldehydic and dialdehydic structures, including the main derivatives oleacin and oleochantal. The main result of these transformations is a strong increase of the complexity of the phenolic fraction, passing from the olive fruit to the virgin oils. Recently, some authors pointed out the need to quantify not only hydroxytyrosol, but also total tyrosol, for the calculation of the olive oil polyphenols to apply to EFSA health claim [12]. Furthermore, the use of mass detectors has helped to improve the knowledge on the phenolic composition in EVOOs, allowing the identification of the presence of lignans as pinoresinol and acetoxypinoresinol [13], which is more than ten years after the first works published on the detection of the secoiridoidic compounds [14]. Even by applying alternative methods to the widely used HPLC-DAD determination, such as capillary electrophoresis [15], the analytical problem of obtaining a satisfactory chromatographic resolution of the secoiridoidic fraction in EVOOs remains so far unresolved. Another reason that increases the complexity of this problem is the chemical instability of the main secoiridoidic derivatives in EVOOs that undergo hydrolysis over time, producing elenolic acid and simple phenols as tyrosol (Tyr) and hydroxytyrosol (OH-Tyr) [16]. This spontaneous phenomenon is also used to evaluate the freshness of the olive oil by measuring the percentage of hydrolysis (calculated as sum of the two simple phenols on the total phenols, that increases in unfresh samples [17,18]).

The first method proposed and applied to simplify the study of the secoiridoidic fraction in EVOOs, was the application of an acidic hydrolysis by H_2_SO_4_ of the total phenolic extract, developed in order to improve the accuracy of the measure of the total hydroxytyrosol and tyrosol content [17,18]. A similar approach was successively proposed for developing the hydrolysis procedure with HCl at room temperature directly on the olive oil as such, and not on the phenolic extract [19]. A further alternative approach to determine the two phenyl ethyl alcohols, using acetyl chloride for the hydrolysis, and a successive derivatization, followed by a determination in GC, has been also proposed [20]. Some researches aimed at simplification of the phenolic profiling via acidic hydrolysis of complex forms has comparatively examined the first two protocols [21,22,23]. Mastralexi et al. (2014) [21,23] concluded that both these two procedures are equally effective and adequate for the purpose, however, provided evidences about the superiority of the method determining total hydroxytyrosol and tyrosol content in the hydrolysate of the polar fraction, rather than directly on the oil [17]. Indeed, this latter method included the possibility of evaluating the total phenols before hydrolysis, according to the IOC (International Olive Council) official method [24] and, recently, it has been also validated [25].

The possibility of having available alternative and simpler procedures applicable for the evaluation of the phenolic fraction opens up the possibility of increasing the analyzed samples which improve the accuracy of the results and to control the quality of the EVOOs, both fresh and after storage.

The main objective of this work was to investigate the phenolic fraction in EVOOs which assists in the application of the EFSA health claim based on polyphenol content, by applying acidic hydrolysis followed by an HPLC-DAD determination. To this aim, the following steps were performed: (i) Extraction of total phenolic compounds by the IOC method; (ii) comparison of different hydrolytic procedures and selection of the more suitable ones for the determination of total hydroxytyrosol and tyrosol; (iii) validation and application of the selected procedures on one hundred oils; (iv) comparison of total phenolic content before and after the acidic hydrolysis to indirectly investigate the secoiridoidic precursors.

## 2. Results and Discussion

### 2.1. Method Optimization and Validation

To facilitate the application of the EFSA health claim on olive oil polyphenols, it is necessary to apply simple analytical procedures to better investigate the secoiridoidic fraction of virgin olive oils. The objective is mainly to distinguish between derivatives of oleuropein and ligstroside, which are hydroxytyrosol and tyrosol respectively, and which are mainly generated by the enzyme β-glucosidase, which removes the glucose moiety from the secoiridoidic ring during the milling process. Tyrosol and hydroxytyrosol are also present in other phenolic compounds of olive fruits like verbascoside [26]), nuzhenide and nuzhenide oleoside [27], but these molecules are not recovered in extracted oils, thus the only sources of these simple phenols in the oils are the abovementioned derivatives of oleuropein and ligstroside. The other class of phenols recovered in the oils in non-negligible amounts is lignans [28], in whose structure, the moieties of tyrosol and hydroxytyrosol are not present. Previous results also showed that a part of the lignans underwent a degradation process during the acid hydrolysis by sulfuric acid at 80 °C [17].

**Figure 1:**
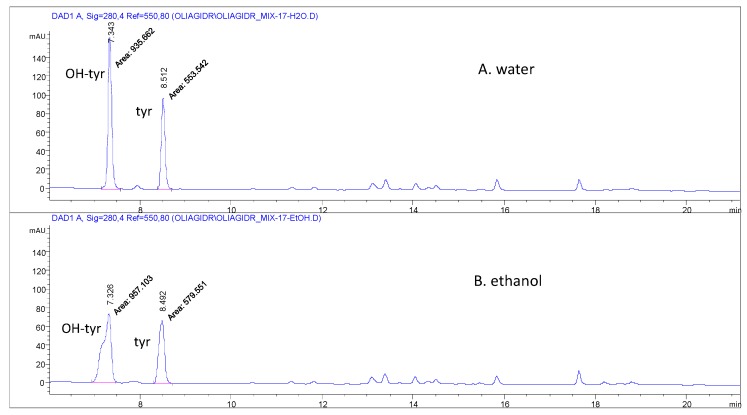
HPLC-DAD profiles at 280 nm of the MIX-17 oil sample after hydrolysis: (**A**), the hydrolysis was quenched using water; (**B**), the hydrolysis was quenched using ethanol.

The appropriate procedure was selected after some preliminary tests were carried out which tested several hydrolytic methods in different acidic media. After hydrolysis, this study applied a new chromatographic method that allowed two well separated peaks for tyrosol and hydroxytyrosol, saving elution solvents and the time of analysis. The results were compared in terms of amounts of tyrosol and hydroxytyrosol obtained by HPLC-DAD from the same sample which constituted by a blend of five EVOOs (MIX-17). These tests took into account several possible modifications of the method previously proposed and applied [17,18]. Firstly, this study evaluated that the use of water instead of ethanol for quenching the hydrolytic reaction after 2 h at 80 °C is preferable. Indeed, Figure 1 shows better shape and resolution for the peaks of tyrosol and hydroxytyrosol when the injected sample is in water. This is because of the distortion effect on the peaks given by the percentage of ethanol in the dissolving medium, which is not entirely compatible with the initial gradient and flow rate. To avoid a distortive effect on the chromatographic peaks, water was added in the final step of the process to stop the hydrolysis carried out on all the 108 EVOOs.

**Figure 2:**
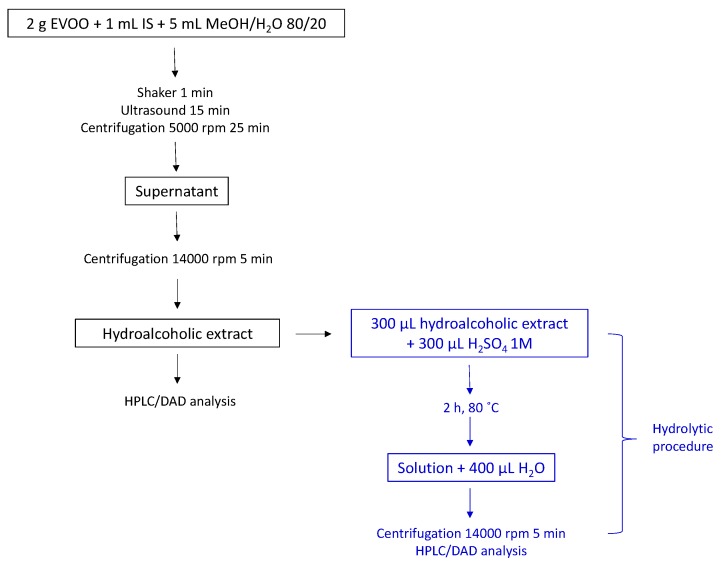
Summary of the proposed method in two successive steps: total phenolic extraction is in black and acidic hydrolysis of the methanol/aqueous extract is in blue.

Following this, several hydrolytic methods in different acidic media were compared. The extracts were prepared in the same day and analyzed immediately after the hydrolysis. According to the results in Table 1, the method previously proposed and applied [17,18], and recently validated [25], has been confirmed as the more suitable for the purposes of this study (line 20, Table 1). It uses the typical phenolic extract obtained by the IOC method using MeOH:H_2_O 80:20 for extraction and sulfuric acid at 80 °C for 2h for hydrolysis, and gives results similar to the same method but using EtOH:H_2_O 80:20 for extraction. The choice of the former method was completed as it allows two results with only one extraction—the total content of phenols according to the IOC official method and the total content of tyrosol and hydroxytyrosol after acidic hydrolysis (Figure 2). The choice of MeOH:H_2_O 80:20 as the best extractive mixture is also in agreement with a recent study [29]. Nevertheless, since ethanol is less toxic than methanol and the use of EtOH:H_2_O 80:20 gives results similar to MeOH:H_2_O 80:20, the choice of the former mixture could be taken into account when the total content of phenols by the official IOC method is not of interest, and data of total content of tyrosol and hydroxytyrosol is the only objective.

The next step was the method validation according to the International Organization for Standardization (Rif- ISO3534-1, 1993), defining the range of linearity, as well as limits of detection (LOD), limits of quantification (LOQ), trueness and precision. The parameters for validation summarized in Table 2 show a good linearity of calibration for the two analytes (R^2^) in the same range (1.0–150.0 mg/kg). For tyrosol, regarding accuracy, trueness was confirmed within the range of 80–120% and precision was assessed with cv% < 5% at both low and high concentrations. For hydroxytyrosol, the slope and intercept are not reported in Table 2. In fact, this study obtained diverse values of these parameters over time, due to a low stability of the stock standard solution of this molecule. This study also confirmed the instability of this molecule by analysis of its aqueous and hydroalcoholic solutions over time, which is in agreement also with our previous studies [30]. Due to working with the calibration line which was built on the same day as preparation of the spiked samples, the authors obtained precision with cv% < 5% at both low and high concentrations, and trueness of 115.7% at high concentration and 121.5% at low concentration. Another aspect to emphasize is that this latter molecule as a pure standard is also much more expensive than tyrosol. For these reasons, it was proposed to use only tyrosol for building the calibration curve, applying the correct multiplicative factor (0.65) needed for a more accurate determination of hydroxytyrosol which was already applied to determine the phenolic composition of several extracts from *Olea europaea* L. [30,31].

Finally, the effectiveness of the hydrolytic procedure was confirmed by evaluation in sixtuplicate of hydroxytyrosol recovered after hydrolysis of solutions prepared with the pure standard of oleuropein at both low (17 mg/L, that is 4.85 mg/L of hydroxytyrosol equivalent) and high (170 mg/L, that is 48.5 mg/L of hydroxytyrosol equivalent) concentrations. The results showed a recovery of 98.0 ± 3.2% at low concentration and of 104.5 ± 1.2% at high concentration with respect to the added amount.

### 2.2. Application of the Method

**Figure 3:**
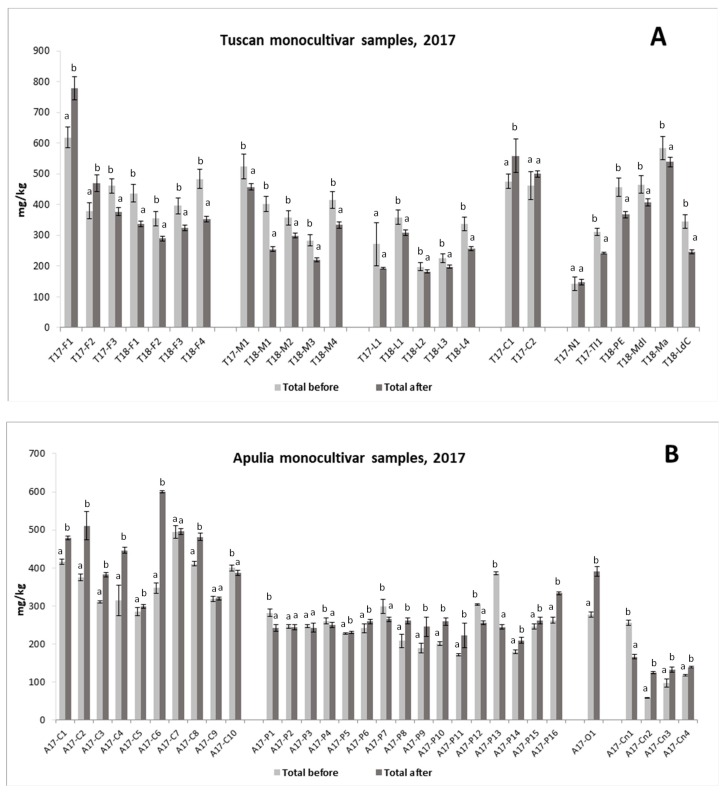
Total phenols evaluated by the IOC method (total before) and as a sum of OH-Tyr +Tyr calculated after the acidic hydrolysis (total after) in monocultivar samples; data are the mean of triplicates; (**A**) oils produced in Tuscany, (**B**) oils produced in Apulia in agreement with Table 3.

The validated method was successively applied to evaluate and compare the phenolic amount of 108 virgin olive oils (Table 3) determined before (according to the IOC procedure) and after the acidic hydrolysis. After hydrolysis, the amount of the other phenols is negligible (Figure 1), thus the total phenolic content was expressed in mg/kg as a sum of hydroxytyrosol and tyrosol (Figure 3A,B). Moreover, in order to provide useful information according to the health claim, the mean values of tyrosol and hydroxytyrosol after hydrolysis and of total phenols before hydrolysis were calculated and expressed in mg/20g_oil_ (Table 4A,B).

The results for monocultivar samples from 2017 are reported in Figure 3 for both Tuscan (3A) and Apulian (3B) oils. Regarding the comparison of the content of phenols before and after hydrolysis, a specific trend was not highlighted and similar values were obtained for most of the samples in both cases. The samples from Frantoio (3A) and Coratina (3B) cultivars showed, on average, the highest concentration of total phenols, while samples from Peranzana (3B) were those with more similar amounts before and after hydrolysis. Table 4 highlights the minimum, maximum and average amounts obtained for the oils produced in 2017 and 2018 before and after hydrolysis. These values give a picture of the phenolic content measured with each of the two analytical approaches, and confirm the absence of a unique trend in the results—the mean content was higher after hydrolysis for Tuscany oils and, on the opposite end, the highest amounts were before hydrolysis for the Apulian oils. The tables also show that only some of all the oils reached the minimum amount of 5mg/20mL_oil_ requested by the EFSA for the application of the health claim. In particular, keeping into account data obtained after hydrolysis, 7 Tuscany samples and 6 Apulian samples showed values below this threshold.

### 2.3. Comparison Between the Content of Tyrosol and Hydroxytyrosol after Hydrolysis

In order to promote the application of the EFSA health claim and to better define the antioxidant potential of the analyzed EVOOs, the percentage of hydroxytyrosol, characterized by the presence of a catechol structure absent in the tyrosol molecule, was calculated after the acidic hydrolysis on the sum of the two phenyl-ethyl alcohols. This evaluation is a simple way to distinguish between the pool of secoiridoids derived from ligstroside and those obtained from oleuropein produced after enzymatic hydrolysis during the milling process. This simple information is very difficult to be extrapolated by HPLC-DAD analysis due to the complex chromatographic profile obtained when applying the IOC method [24], which is mainly due to the presence of several isobaric derivatives of secoiridoids.

**Figure 4:**
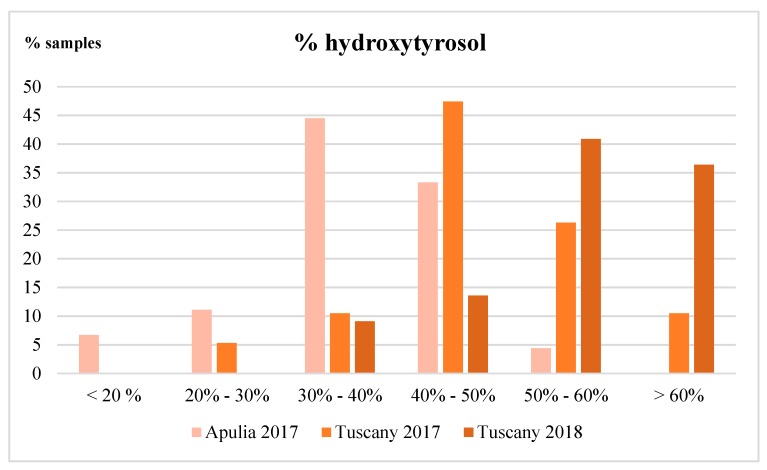
Evaluation of the percentage of hydroxytyrosol on the sum of total hydroxytyrosol + tyrosol. The chart reports the percentage of samples with different ranges of percentage of hydroxytyrosol for the sample from Apulia 2017, Tuscany 2017 and Tuscany 2018.

The percentages of hydroxytyrosol obtained for all the analyzed oils are reported in Figure 4, where very different ranges of values are for the oils from Apulia 2017, Tuscany 2017 and Tuscany 2018. Most of the Apulian oils showed percentages below 50% and a few of them were even below 20%. These results, obtained from 45 samples, highlighted a predominance of tyrosol in the phenolic fraction in almost all the Apulian oils. Regarding the Tuscan oils from 2017, most of the samples showed again a predominance of tyrosol (approx. 63%), but, in this case, a larger number of samples was in the range of 40%–50% of hydroxytyrosol. On the contrary, the hydroxytyrosol was largely predominant in the Tuscan samples of 2018 (44 EVOOs), with values higher than 50% in approx. 71% of samples. As Tuscan oils from the two years were almost all from the same geographical areas and mills, it can be hypothesized that climate plays an important role in the biosynthesis of different secoiridoids in the fruit and, consequently, on the final amount of hydroxytyrosol in the oils. Nevertheless, these findings can be considered as preliminary, as this aspect has not been investigated sufficiently in the literature to date. Further studies, mainly focused on monocultivar oils, are needed to confirm the effect of climate changes on the final composition of the secoiridoidic fraction in the virgin olive oils.

Recently, other authors discussed the need to sum the tyrosol derivatives to those from oleuropein to calculate the minimum amount of phenols required by EFSA [2] to apply the health claims [12]. To date, only a small number of works are available with data regarding the relative amount of tyrosol and hydroxytyrosol in the EVOOs. From this research which applied acidic hydrolysis [17,18] or a NMR determination [32,33,34], it emerged that a large part of the samples contain tyrosol in higher percentages. The application of this hydrolytic procedure on a large number of samples, will be useful to provide a more realistic assessment of the composition of a secoiridoid pool in virgin olive oils.

## 3. Materials and Methods

### 3.1. Chemicals

Formic acid, sulfuric acid (95.0%–98.0%), methanol and acetonitrile of HPLC grade, methanol and ethanol of analytical grade were obtained from Sigma Aldrich (Steinheim, Germany). Ultrapure water was obtained from the Milli-Q-system (Millipore SA, Molsheim, France). Tyrosol (≥98%), hydroxytyrosol (≥98%) and oleuropein (≥98%) were purchased from Extrasynthèse (Genay, France).

Standard stock solutions were prepared as follows: Oleuropein 175 mg/L in MeOH:H_2_O 80:20, hydroxytyrosol 1500 mg/L in MeOH:H_2_O 80:20 *v*/*v* and tyrosol 1,500 mg/L in MeOH:H_2_O 80:20. These stock solutions were stored at −18 °C and warmed at room temperature when used for preparing the diluted solutions for calibration. These latter were prepared daily for tyrosol and hydroxytyrosol, starting from different aliquots of stock solutions for obtaining diluted solutions in the range of 1–150 mg/L. This working range was selected based on the typical contents of tyrosol and hydroxytyrosol that can be expected to be found in virgin olive oils.

### 3.2. Oil Samples

The extra virgin olive oil samples of this study have been collected from different Tuscan (63 samples) and Apulian (45 samples) olive mills from the olive oil campaigns of 2017 and 2018. As listed in Table 3, samples were either monocultivar or blend. This study also prepared a mixture (MIX-17) constituted of 5 samples (A17-C3, A17-C6, A17-C10, A17-P10 and A17-O1) which were selected to take into account a maximum variability in terms of geographical area of production and cultivar.

### 3.3. Phenol Extraction and Acidic Hydrolyses

Phenolic compounds from olive oils were extracted and analyzed according to the IOC official method [24]. Phenolic compounds were extracted with MeOH:H_2_O 80:20 *v*/*v*, and immediately analyzed. The chromatographic analysis was performed according to the IOC method [24], using an HP 1100 system provided with a quaternary pump and coupled to a diode array detector (Agilent Technologies, Santa Clara, CA, USA). The column was a SphereClone ODS (2), 5μm, 250 × 4.6 mm id. Elution was obtained by H_2_O (at pH 3.2 by formic acid), acetonitrile and methanol as eluents, applying the gradient reported in the IOC method, with a flow rate of 1 mL min^−1^. The injection volume was 20 μL. The areas were registered at 280 nm, with syringic acid as the internal standard. The content of phenolic compounds was expressed as mg_tyr_/kg_oil_.

Preliminarily, a mix of five EVOOs (MIX-17) was used to compare the performance of different hydrolytic procedures in acidic media, using both sulfuric and hydrochloride acids. The different tested experimental conditions are summarized in Table 1. After selecting the best protocol, phenolic extracts were prepared according to the IOC method using methanol/water (80:20 *v*/*v*).

After acidic hydrolysis, the hydrolyzed extracts were analyzed by a different chromatographic method selected to reduce the analysis time and the flow rate with a solvent saving. Furthermore, the use of methanol, known to be toxic, was avoided and acetonitrile and H_2_O (at pH 3.2 by formic acid) were selected as eluents. Consequently, all the hydrolyzed samples were analyzed with an HP1200 liquid chromatograph coupled to a diode array detector (Agilent Technologies, Santa Clara, CA, USA) and a 150 × 3 mm (5 μm) Gemini RP18 column (Phenomenex, Torrance, CA, USA). The flow rate was 0.4 mL min^−1^ and the total analysis time was 22 min. A linear gradient was applied starting from 95% to 70% A in 5 min, to 50% A in 5 min, to 98% B in 5 min with a final plateau of 5 min.

### 3.4. Quantification of Phenolic Content

Total phenolic content (such as the original and oxidized derivatives of oleuropein and ligstroside, lignans, flavonoids and phenolic acids) before acidic hydrolysis was evaluated according to the official IOC method [24]. The content of total tyrosol and hydroxytyrosol after hydrolysis was evaluated using the tyrosol standard (purity grade 98%) to prepare the calibration curves at 280 nm. During method development performed at the beginning of the study (crop 2017), six different calibration curves in six different days were built for estimating precision and accuracy (see also 3.5 paragraph) and all of them were fitted to simple linear calibration using Microsoft Excel 2010. A new calibration curve was then built for the analysis of samples from 2018. As the content of hydroxytyrosol is overestimated to 35% when evaluated using the calibration curve built with tyrosol as standard [30], the following formula has been applied to obtain the accurate amount of total hydroxytyrosol: mg_OH-tyr_ = mg_tyr_ × 0.65 [21]. All data were expressed as mg/kg_oil_.

### 3.5. Validation Procedure

The quality parameters for the validation of the hydrolytic procedure are summarized in Table 2: Range of linear calibration, sensitivity (slope of the calibration straight-line), limits of quantification (LOQ), limits of detection (LOD), linearity (in terms of R^2^) and accuracy (in terms of trueness and precision), are reported for the two external standards used for the quantitative evaluation. The LOQ and the LOD were calculated as the lowest concentration level of the calibration line with precision as CV% ≤ 20% and accuracy in the 80%–120% recovery range and as one third of the LOQ, respectively. The highest point with accuracy in the 80%–120% recovery range and precision as CV% ≤ 20%, both were calculated on the six replicates of the respective calibration levels selected as the upper end of the calibration line. The squared adjusted regression coefficient allowed confirming the linearity of the calibration, while accuracy was evaluated in terms of trueness and precision [35,36]. Both these parameters were assessed using six replicates of two level spiked samples. The first, at low concentration, was 1.15*LOQ, while the second, at high concentration, was at the penultimate point of the calibration curve. Precision was calculated as CV% = SD × 100/Cm and the trueness in terms of apparent recovery R, calculated as R = (Cm × 100)/Cref, where SD was the standard deviation, Cm the mean value and Cref the reference value. The selectivity of the method was easily verified by the optimal chromatographic resolution between hydroxytyrosol and tyrosol (Figure 1).

### 3.6. Statistical Analysis

Data in Table 1 and Figure 3 are the mean of three determinations. One-way ANOVA and F-test (*p* < 0.05) were performed using Microsoft Excel statistical software for evaluating statistical significance, then the means were compared by Fisher’s LSD test using the DSAASTAT excel^®^ VBA macro, version 1.1 (Onofri, A.; Pisa, Italy, 2007).

## 4. Conclusions

The EFSA health claim regarding polyphenols of virgin olive oil, when reported on labels, can be a useful legislative tool allowing the consumer to recognize the highest quality segment within the product category of extra virgin olive oils. However, to date, only a very low percentage of producers apply this claim on the label, and suitable analytical methods to overcome this issue are still missing.

This work proposed and applied an acidic hydrolysis on phenolic extracts for assisting in the application of the EFSA health claim regarding olive oil polyphenols. The proposed method is suitable to determine the total content of tyrosol and hydroxytyrosol, starting from the methanol extract according to the IOC protocol, which was recently confirmed as the best extractive procedure for the recovery of the total phenols from virgin olive oils. Furthermore, this study proposed the use of a suitable corrective factor for the accurate evaluation of hydroxytyrosol using only the calibration curve of tyrosol.

The hydrolysis method was applied to more than one hundred EVOOs produced in two Italian regions in two successive years. Overall, similar values of total phenols were obtained before and after hydrolysis for the most of the samples and data after hydrolysis indicated that approximately 75% of samples reached the minimum amounts of phenols requested by the EFSA for the application of the health claim. A predominance of tyrosol on hydroxytyrosol was highlighted in almost all the Apulian oils and for the Tuscany samples from 2017 crop, while for those from the 2018 crop, this trend was not confirmed, suggesting a role of climate in the biosynthesis of the secoiridoidic pattern in the virgin oils.

This approach allowed a simple and accurate measure of the total hydroxytyrosol and tyrosol, which is a pre-requisite to correctly apply the EFSA health claims. Finally, the availability in future data regarding the evolution of the phenolic fraction after acidic hydrolysis over time could provide useful regarding the current shelf life, at least for high quality EVOOs, thereby, helping producers to highlight the nutraceutical potential when applying the EFSA health claims.

## Figures and Tables

**Table 1:** Evaluation of the maximum recovery in terms of OH-Tyr and Tyr after different hydrolysis methods in acidic media; the analyses were applied to the MIX-17 sample; the data are a mean of triplicates expressed as mg/kg. Under the column, condition, the authors specified (i) the extraction solvent (EtOH = EtOH:H_2_O 80:20; MeOH = MeOH:H_2_O 80:20), (ii) the acid used for hydrolysis (HCl = HCl 2 M; H_2_SO_4_ = H_2_SO_4_ 1M), (iii) the hydrolysis temperature and iv) the hydrolysis time. In each column, different letters indicate statistical differences at p 0.05.

n°	Conditions	Hydroxytyrosol	Tyrosol
1	EtOH HCl 25 °C-2h	135.3 ± 6.1 c	120.1 ± 6.1 c
2	EtOH HCl 25 °C-4h	176.6 ± 3.5 g	163.0 ± 3.4 fg
3	EtOH HCl 25 °C-6h	198.8 ± 0.6 i	187.7 ± 0.8 ij
4	EtOH HCl 25 °C-24h	206.2 ± 1.9 jk	197.5 ± 1.8 kl
5	EtOH HCl 80 °C-2h	158.5 ± 1.0 d	203.1 ± 0.2 lm
6	EtOH H_2_SO_4_ 25 °C-2h	119.0 ± 6.8 b	103.3 ± 5.9 b
7	EtOH H_2_SO_4_ 25 °C-4h	167.6 ± 1.8 ef	147.8 ± 2.4 e
8	EtOH H_2_SO_4_ 25 °C-6h	188.3 ± 0.4 h	166.5 ± 3.0 g
9	EtOH H_2_SO_4_ 25 °C-24h	203.8 ± 0.4 ijk	183.9 ± 0.9 hi
10	EtOH H_2_SO_4_ 80 °C-2h	216.2 ± 1.5 l	205.1 ± 0.4 m
11	MeOH HCl 25 °C-2h	110.2 ± 8.8 a	92.3 ± 7.4 a
12	MeOH HCl 25 °C-4h	178.5 ± 2.7 g	159.8 ± 2.4 f
13	MeOH HCl 25 °C-6h	200.3 ± 0.4 ij	180.9 ± 1.1 h
14	MeOH HCl 25 °C-24h	205.7 ± 2.1 jk	192.0 ± 1.9 jk
15	MeOH HCl 80 °C-2h	172.6 ± 0.7 fg	201.5 ± 1.0 lm
16	MeOH H_2_SO_4_ 25 °C-2h	120.0 ± 9.5 b	99.5 ± 7.7 b
17	MeOH H_2_SO_4_ 25 °C-4h	165.3 ± 2.3 e	140.9 ± 2.7 d
18	MeOH H_2_SO_4_ 25 °C-6h	190.4 ± 4.0 h	166.0 ± 6.8 g
19	MeOH H_2_SO_4_ 25 °C-24h	205.9 ± 1.0 jk	179.1 ± 1.3 h
20	MeOH H_2_SO_4_ 80 °C-2h	209.4 ± 1.9 k	197.6 ± 3.4 kl

**Table 2:** Quality parameters for method validation regarding the external standards used for the quantitative evaluation.

Phenolic Compound	rt (min)	Range of Linear Calibration (µg/mL)	Slope	Intercept	R^2^_adj_	LOD (µg/mL)	LOQ (µg/mL)	Low Concentration Trueness (R%)	Low Concentration Precision (CV%)	High Concentration Trueness (R%)	High Concentration Precision (CV%)
**Tyrosol**	8.5	1.0–150.0	1601.6 ± 13.9	5.3 ± 4.2	1.0000	0.33	1.0	89.7	2.4	98.0	1.0
**Hydroxytyrosol**	7.3	1.0–150.0	-	-	0.9998	0.33	1.0	121.5*	1.6	115.7	1.9

rt, retention time; R^2^, squared regression coefficient; * values of accuracy out of the range 80%–120%.

**Table 3:** Analyzed oils produced in Tuscan (T) and Apulian (A) olive mills. F, Frantoio; No, Nocellara; C, Coratina; TI, Tonda Iblea; M, Moraiolo; L, Leccino; P, Peranzana; Cn, Cellina di Nardò; O, Ogliarola; PE, Pendolino; MdI, Madonna dell’Impruneta; MA, Maurino; LdC, Leccio del Corno; B, blend.

Apulia 2017	Cultivar	Tuscany 2017	Cultivar	Tuscany 2018	Cultivar
A17-C1	Coratina	T17-F1	Frantoio	T18-F1	Frantoio
A17-C2	Coratina	T17-B1	Blend	T18-L1	Leccino
A17-P1	Peranzana	T17-B2	Blend	T18-M1	Moraiolo
A17-P2	Peranzana	T17-B3	Blend	T18-B1	Blend
A17-P3	Peranzana	T17-N1	Nocellara	T18-B2	Blend
A17-P4	Peranzana	T17-C1	Coratina	T18-M2	Moraiolo
A17-P5	Peranzana	T17-B4	Blend	T18-F2	Frantoio
A17-P6	Peranzana	T17-B5	Blend	T18-L2	Leccino
A17-P7	Peranzana	T17-B6	Blend	T18-F3	Frantoio
A17-P8	Peranzana	T17-C2	Coratina	T18-L+M	Leccino + Moraiolo
A17-P9	Peranzana	T17-TI1	Tonda Iblea	T18-L+F	Leccino + Frantoio
A17-P10	Peranzana	T17-L1	Leccino	T18-B3	Blend
A17-P11	Peranzana	T17-F2	Frantoio	T18-B4	Blend
A17-P12	Peranzana	T17-F3	Frantoio	T18-L3	Leccino
A17-B1	Blend	T17-M1	Moraiolo	T18-PE	Pendolino
A17-B2	Blend	T17-B7	Blend	T18-L4	Leccino
A17-B3	Blend	T17-B8	Blend	T18-MdI	Madonna dell’Impruneta
A17-C/O1	Coratina/Ogliarola	T17-B9	Blend	T18-M3	Moraiolo
A17-C/O2	Coratina/Ogliarola	T17-B10	Blend	T18-F4	Frantoio
A17-Cn1	Cellina di nardò			T18-M4	Moraiolo
A17-Cn2	Cellina di nardò			T18-B5	Blend
A17-Cn3	Cellina di nardò			T18-MA	Maurino
A17-Cn4	Cellina di nardò			T18-LdC	Leccio del Corno
A17-C1	Coratina			T18-B6	Blend
A17-C2	Coratina			T18-B7	Blend
A17-C3	Coratina			T18-B8	Blend
A17-P1	Peranzana			T18-B9	Blend
A17-P2	Peranzana			T18-B10	Blend
A17-B1	Blend			T18-B11	Blend
A17-B2	Blend			T18-B12	Blend
A17-B3	Blend			T18-B13	Blend
A17-P3	Peranzana			T18-B14	Blend
A17-P4	Peranzana			T18-B15	Blend
A17-C/O1	Coratina/Ogliarola			T18-B16	Blend
A17-C4	Coratina			T18-B17	Blend
A17-C/O2	Coratina/Ogliarola			T18-B18	Blend
A17-C/O3	Coratina/Ogliarola			T18-B19	Blend
A17-B4	Blend			T18-B20	Blend
A17-C5	Coratina			T18-B21	Blend
A17-B5	Blend			T18-B22	Blend
A17-C6	Coratina			T18-B23	Blend
A17-C7	Coratina			T18-B24	Blend
A17-O1	Ogliarola			T18-B25	Blend
A17-C8	Coratina			T18-B26	Blend
A17-B6	Blend				

**Table 4:** Evaluation of the mean values before and after acidic hydrolysis was applied to 64 extra virgin olive oils produced in Tuscany and Apulian regions in 2017 (**A**) and in Tuscany in 2018 (**B**).

**A**	**After Hydrolysis (mg/20goil)**			**Before Hydrolysis (mg/20goil)**
	**Hydroxytyrosol**	**Tyrosol**	**Tyrosol + Hydroxytyrosol**	**Total phenols**
**max**	8.90 ± 0.67	7.24 ± 0.59	15.56 ± 1.09	12.36 ± 1.39
**min**	0.29 ± 0.01	1.38 ± 0.01	2.48 ± 0.01	1.17 ± 0.02
**mean**	2.93 ± 0.11	4.08 ± 0.12	7.00 ± 0.22	6.34 ± 0.29
**B**	**After Hydrolysis (mg/20goil)**			**Before Hydrolysis (mg/20goil))**
	**Hydroxytyrosol**	**Tyrosol**	**Tyrosol + Hydroxytyrosol**	**Total phenols**
**max**	6.92 ± 0.20	4.06 ± 0.12	10.78 ± 0.31	11.69 ± 0.75
**min**	0.63 ± 0.02	1.11± 0.03	1.76 ± 0.05	3.36 ± 0.22
**mean**	3.23 ± 0.09	2.45 ± 0.07	5.68 ± 0.16	6.89 ± 0.44

## Supplementary Materials

The following are available online https://www.mdpi.com/1420-3049/24/11/2179/s1, Table S1: classification of Health Claims (EU Regulation 1924/2006). Table S2: list of permitted health claims for olive oil and the relative conditions of use. Table S3: SWOT analysis used to evaluate the need for health claims for the EVOO producers.
