# Supplementary material for: The EFSA Health Claim on Olive Oil Polyphenols: Acid Hydrolysis Validation and Total Hydroxytyrosol and Tyrosol Determination in Italian Virgin Olive Oils
Source: Molecules. 2019 Jun 10;24(11):2179. doi: 10.3390/molecules24112179 (PMC6600398; doi:10.3390/molecules24112179)
Supplement: Supplementary file 1 [file molecules-24-02179-s001.pdf]

SUPPLMENTARY MATERIAL

Foods are not bought for the simple purpose of satisfying a primary need, appeasing hunger and eating, but with the full awareness of the link between food and health. This awareness stimulates in the consumer the need to know the composition of what he introduces into his body, looking for guarantees on the absence of harmful molecules and hoping for the presence of components with beneficial and functional action.

For companies, the possibility of identifying an element of distinctiveness in the health effects of food represents a useful marketing tool. Even the olive oil sector does not avoid this market logic.

The Regulation recognizes two types of claim:

- nutritional claims, any indication which states, suggests or implies that a food has particular beneficial nutritional properties due to the energy (calorific value) it provides or does not provide, or the nutrients or other substances it contains or does not contain;

- and health claims, any statement about a relationship between food and health concerning the existence of a relationship between a food or its component and health, based on scientific evidence and can be easily understood by consumers (Table S1)

Table S1. Classification of Health Claims (EU Regulation 1924/2006)

Types of Health Claims	
'Function Health Claims'**(or Article 13 claims)**	- Relating to the growth, development and functions of the body<br>- Referring to psychological and behavioral functions<br>- On slimming or weight-control
'Risk Reduction Claims' **(or Article 14(1)(a) claims) on reducing a risk factor in the development of a disease.**	"Plant stanol esters have been shown to reduce blood cholesterol. Blood cholesterol is a risk factor in the development of coronary heart disease"
'Claims referring to children's development' **(Article 14(1)(b) claims)**	"Vitamin D is needed for the normal growth and development of bone in children"

Nutrition and health claims must not be false, ambiguous, misleading, or give rise to doubts about other foods; they must not encourage excessive consumption of a particular food or to directly or indirectly state that a balanced and varied diet cannot generally provide adequate quantities of all nutrients arousing or exploit fears in the consumer.

Table S2 shows the permitted health claims for olive oil.

Table S2. List of permitted health claims for olive oil and the relative conditions of use.

Substance	Claim	Conditions of use of the claim
**Olive oil polyphenols**	Olive oil polyphenols contribute to the protection of blood lipids from oxidative stress	The claim may be used only for olive oil which contains at least 5 mg of hydroxytyrosol and its derivatives (e.g. oleuropein complex and tyrosol) per 20 g of olive oil. In order to bear the claim information shall be given to the consumer that the beneficial effect is obtained with a daily intake of 20 g of olive oil.
**Oleic acid**	Replacing saturated fats in the diet with unsaturated fats contributes to the maintenance of normal blood cholesterol levels. Oleic acid is an unsaturated fat.	The claim may be used only for food which is high in unsaturated fatty acids, as referred to in the claim high unsaturated fat as listed in the Annex to Regulation (EC) No 1924/2006 and subsequent amendments A claim that a food is high in unsaturated fat, may only be made where at least 70 % of the fatty acids present in the product derive from unsaturated fat under the condition that unsaturated fat provides more than 20 % of energy of the product.
**Vitamin E**	Vitamin E contributes to the protection of cells from oxidative stress	The claim may be used only for food which is at least a source of vitamin E as referred to in the claim source of vitamin e as listed in the Annex to Regulation (EC) No 1924/2006 and subsequent amendments.
**Monounsaturated**	Replacing saturated fats with	The claim may be used only for food which is high in

**and/or polyunsaturated fatty acids**	unsaturated fats in the diet has been shown to lower/reduce blood cholesterol. High cholesterol is a risk factor in the development of coronary heart disease	unsaturated fatty acids, as referred to in the claim high unsaturated fat as listed in the Annex to Regulation (EC) No 1924/2006 and subsequent amendments. The claim may only be used on fats and oils

Analyzing Table S2, it can be seen that while the claims of tocopherols and fatty acids are applicable to all the categories of olive oils, the claim of polyphenols is suitable only for the extra virgin category because the refining processes determine their removal from the olive oils as well from other virgin oils as cold pressed grape seed oils.

Since the phenolic molecules are of hydrophilic nature, the olive millers know well how difficult it is to obtain oils rich in these antioxidants, whose presence in the oil is a function, in addition to the varietal origin, of agronomic practices (pruning, fertilizing, irrigation and phytosanitary treatments) and technological aspects. Moreover, it is well known that oil yield and content in phenols are aspects that are always in antithesis in the production of extra virgin olive oil.

An empirical observation of the market makes it clear that despite the wide range of advantages deriving from the use of claims, few producers or marketing companies, 7 years after the publication of the EU regulation, systematically use one of the most advantageous marketing tools in the agri-food sector. The SWOT analysis used to evaluate the need for health claims for the EVOO producers is reported in Supplementary Table S3.

Table S3. SWOT analysis used to evaluate the need for health claims for the EVOO producers.

**Strengths**	**Weaknesses**
- Strong ability to add value to products<br>- Use of already authorized health claims<br>- Strong ability to differentiate products<br>- Strong ability to resolve the gap of the of "asymmetry information"<br>- To communicate and to influence the eating culture<br>- Strong credibility of the claim deriving from the evaluation of EFSA panel of expert	- Lack of skilled employees with specialized knowledge (e.g. about legal requirements)<br>- Focus on just authorized health claims<br>- A tool to predict the permanence of the claim validity during the shelf life of the product lacks<br>- Knowledge about health claims on EVOO and their impact on purchase attitude and consumption behaviour of consumers is still limited.<br>**- Regard to the claim of polyphenols, an official method of analysis lacks**<br>**- Regard to the claim of polyphenols, the analytical description by EFSA of the molecules to be quantified is unclear.**<br>- Regard to the claim of polyphenols, it is difficult to understand for the average consumer.
**Opportunities**	Threats
- Trade inside and outside the EU<br>- Increasing support of EFSA and European Commission<br>- Growing consumer demand for healthy food - Increased segmentation of markets into target groups with specific needs (i.e. elderly people; infants and children, pregnant women; sportsmen)	- Increased power of EVOO bottling firms<br>- Increased competition from emerging producing countries<br>- Aging and declining European population affecting employment and markets

One of the main obstacles to the use of the polyphenol claim is the lack of an official method of analysis to protect the consumer, on the veracity of the claim, and the manufacturer, in the event of verification by the control bodies and in the event of legal dispute for the labeling. At the same time, the possibility to apply the claim is linked to the oil stability over time, which is associated to the natural consumption of the native polyphenols. To this aim also the actual shelf life of eighteen months from bottling has to be re-thought for the segment of virgin olive oils of higher quality.
